# The Response of Governments and Public Health Agencies to COVID-19 Pandemics on Social Media: A Multi-Country Analysis of Twitter Discourse

**DOI:** 10.3389/fpubh.2021.716333

**Published:** 2021-09-28

**Authors:** Lan Li, Aisha Aldosery, Fedor Vitiugin, Naomi Nathan, David Novillo-Ortiz, Carlos Castillo, Patty Kostkova

**Affiliations:** ^1^Centre for Digital Public Health in Emergencies (dPHE), Institute for Risk and Disaster Reduction, University College London, London, United Kingdom; ^2^Department of Information and Communication Technologies, Universitat Pompeu Fabra, Barcelona, Spain; ^3^Institute of Public Health (Alumna), Jagiellonian University, Kraków, Poland

**Keywords:** social media, Twitter, COVID-19, topic modeling, government, public health agencies, public health emergencies

## Abstract

During the COVID-19 pandemic, information is being rapidly shared by public health experts and researchers through social media platforms. Whilst government policies were disseminated and discussed, fake news and misinformation simultaneously created a corresponding wave of “infodemics.” This study analyzed the discourse on Twitter in several languages, investigating the reactions to government and public health agency social media accounts that share policy decisions and official messages. The study collected messages from 21 official Twitter accounts of governments and public health authorities in the UK, US, Mexico, Canada, Brazil, Spain, and Nigeria, from 15 March to 29 May 2020. Over 2 million tweets in various languages were analyzed using a mixed-methods approach to understand the messages both quantitatively and qualitatively. Using automatic, text-based clustering, five topics were identified for each account and then categorized into 10 emerging themes. Identified themes include political, socio-economic, and population-protection issues, encompassing global, national, and individual levels. A comparison was performed amongst the seven countries analyzed and the United Kingdom (Scotland, Northern Ireland, and England) to find similarities and differences between countries and government agencies. Despite the difference in language, country of origin, epidemiological contexts within the countries, significant similarities emerged. Our results suggest that other than general announcement and reportage messages, the most-discussed topic is evidence-based leadership and policymaking, followed by how to manage socio-economic consequences.

## Introduction

Starting in December 2019, the disease caused by the newly isolated coronavirus named COVID-19 spread from the city of Wuhan in China to other countries. COVID-19 is a rapidly transmitted disease spread through respiratory droplets and contact transmission (1). On 30 January 2020, the World Health Organization (WHO) declared that the COVID-19 outbreak constituted a Public Health Emergency of International Concern (PHEIC).

In response to the pandemic, governments and public health institutions adopted various approaches and applied different measures to control the outbreak, such as social distancing, hand hygiene, contact “bubbles,” and self-isolation. Various social network platforms were used to inform people in real-time about the status of the pandemic and government measures, including new restrictions. Government organizations, public health institutions, and world leaders used social media as a primary means of communication to keep the public updated during the pandemic (2, 3).

Many studies have applied topic extraction to analyze social media discourse (4–6). Some of this research explored social media data posted during the pandemic (7–9). Previous work has used several techniques for extracting topics, with text clustering emerging as a key technique,. Both Latent Dirichlet Allocation models (LDA) and K-Means clustering are algorithms which demonstrate good performance in text clustering tasks by using text features. However, in our study a mixed methods methodology was used to provide further context and depth to the findings. An additional vector space-based K-Means clustering algorithm (10) was used to prepare tweets for qualitative analysis. This qualitative element allows researchers to draw conclusions from a public health perspective and to analyze government discourse not possible by quantitative methods alone. The employment of these methodologies makes our study unique in the field of social computing.

Many studies have identified the potential of social media as a source of early warning about pandemics (11–13), a platform for risk communication, as a meter for media and public reaction (14, 15), and to track the spread and source of misinformation (16, 17). Previous studies have also focused on accounts of governments and influential politicians and leaders on social media platforms including Twitter, Facebook, and Weibo. One such qualitative study monitored and analyzed the usage of Twitter by the G7 leaders during the pandemic using content analysis (2). It demonstrated, alongside other findings, that the majority of leaders' tweets can be classified into an “informative” theme (82.8%), followed by “morale boosting” theme (9.4%) and a “political” theme (6.9%) (2). Another study examined the accounts of 143 state leaders on Twitter using regression modeling, to investigate the growth in their number of followers (3). The study showed a substantial increase in the number of followers to these accounts during the pandemic compared to prior periods, which revealed the growing interest of the public for updates from these leaders (3). However, neither of these two studies analyzed the public's tweets in response to their leaders.

Liao et al. conducted a content analysis to analyze 273 posts from 10 Chinese government accounts that were active on Weibo, including both health- and non-health-related accounts (18). Their research showed that the major thematic categories of these accounts were the epidemic status, general information about virus-caused pneumonia, policies, guidelines, and official actions (18). Another study used descriptive statistics and content analysis to analyze 1,215 Weibo posts by 134 of China's Centre for Disease Control and Prevention (CDC) government accounts (19). This study showed that popular health knowledge posts ranked as the most frequently posted topic with about 49.9%, while other topics, such as hygiene, emergency information, citizen consultation, and policy had the least numbers of posts, between 2 and 0.6% (19). Both studies were limited to the content of posts by the accounts and the direct engagement of the public with these posts. The posts of the general public mentioning or addressing these accounts were not included in the content analysis.

To the best of our knowledge, the study conducted by Ramkumar and colleagues has been the only one so far that performed a thematic analysis using both posts from official accounts and comments and responses from the public (20). It reviewed the Facebook pages of three public health authorities: the Centres for Disease Control and Prevention in the United States (CDC), the Ministry of Health (MOH) in Singapore, and Public Health England (PHE) in England. Six major themes were identified using qualitative content analysis: situation updates, preventive measures, public reassurance, disease information, falsehood correction, and “others.” The study found that the top theme for both CDC and PHE was preventive measures. In contrast, most of the MOH posts were situation updates and messages on preventive measures (20).

The studies discussed above were limited to qualitative analysis, using content analysis based on manual coding. This underscores a lack of policy analysis using quantitative methods on a large scale. Policy-related studies so far have exclusively been qualitative, with a lack of data and horizontal comparisons across countries (21–23), especially regarding public reactions and satisfaction, which are hard to quantify. Although some studies attempt to use the data obtained from social surveys to assess the public's response to the policy, results are limited by relatively small sample sizes (24, 25). Social media platforms such as Twitter and Facebook bridge the divide between the public and policy makers and can serve a crucial role in providing data for research. However, quantitative analysis of this important discourse must be performed carefully due to the presence of biases (11, 14). Indeed, one of the key recommendations to deal with these biases is the use of qualitative analysis (26), as employed by this study.

This study is unique in a way it used a mixed-methods methodology—a combination of quantitative and qualitative approaches to analyze and compare social media posts and comments of government and public health agency accounts in the selected countries. We reviewed both official messages as well as the responses from members of the public with the goal of providing recommendations on public health communication during future pandemics and emergency situations.

The study intends to answer one research question related to the usage of Twitter during this pandemic by government agencies and public health agencies: *What were the primary themes of COVID-19- related tweets by governments and public health agencies and the public's reaction toward them?*

## Datasets and Methods

In this section, we will first describe the datasets and then discuss the methodology we used for collecting and analyzing the tweets.

### Description of Datasets

This project started with a dataset collected by the authors, using Twitter Archiving Google Sheet (TAGS) V6.1 (27). This dataset played an important role in helping us to select the most active accounts among government organizations, public health agencies, and national leaders. However, this initial collection was limited due to the restriction of being used only on accounts with over 1,000 followers. This prevented us from gathering replies from many members of the public that do not have this number of followers, but it provided a valuable overview of the themes and topics covered and allowed us to formulate our search criteria for the main data collection. The bulk of the data used in this paper comes instead from a large and multilingual dataset GeoCov19 collected by the Qatar Computing Research Institute (QCRI) (28), which was combined with the TAGS dataset for carrying out the following analysis. In this section, we will present the information and characteristics of each dataset and how we select the study country and Twitter account from the final combined dataset.

#### TAGS Dataset

The TAGS data was collected by the authors during the period of 23 March 2020 to 29 May 2020, with the overall number of tweets at 6,508,227 and retweets at 4,787,271. The Twitter track had two queries to capture a COVID-19 dataset: all tweets containing the hashtag #COVID19 and users who have over 1,000 followers. The peak volume of both the tweets and retweets was in the first week of April; the volume continued to increase until the end of April 2020, and after that, the curve fluctuated. Peak volume days were mapped to the announcement of lockdown measures that had been taken in some countries, such as the UK and Spain (see Table 1). Moreover, the attention to COVID-19 increased significantly in the interval between the end of March to the end of May.

**Table 1:** Selected accounts and the total number of tweets in the final combined dataset.

**Country**	**First lockdown started**	**Organization**	**Twitter account**	**Tweets number (From the combination of TAGS & GeoCov19 datasets)**
United Kingdom (including England, Scotland, Northern Ireland)	23 March	Government of the UK	@govuk	26,120
		Public Health England	@phe_uk	41,748
		First Minister of Scotland	@NicolaSturgeon	61,063
		Public Health Scotland	@P_H_S_Official	2,037
		Scottish Government	@scotgov	35,892
		Department of Health Northern Ireland	@Healthdpt	17,494
		Public Health Agency Northern Ireland	@Publichealthni	15,466
		NI Executive	@Niexecutive	5,602
Spain	15 March	Ministry of Health	@sanidadgob	63,765
		Government of Spain	@desdelamoncloa	33,828
		Instituto de Salud Carlos III	@Saludisciii	8,896
United States	From19 March (California) to 3 April (Georgia); Varies for different states	The White House	@WhiteHouse	31,427
		Centers for Disease Control	@cdcgov	42,781
		Donald J. Trump	@realDonaldTrump	499,567
Canada	17 March	Government of Canada	@ServiceCanada_E	1,009
		Public Health Agency of Canada	@GovCanHealth	20,563
Mexico	1 April	Gobierno de México	@gobmx	1,120
		Presidencia del Gobierno de México	@GobiernoMX	56,299
Brazil (16 states)	7 May	Oswaldo Cruz Foundation	@fiocruz	2,635
	24 March	Government accounts	@SGPresidencia, @govbr, @casacivilbr, @sgovpr	283
Nigeria	24 March	Nigeria Centre for Disease Control	@NCDCgov	33,941
		Nigerian Institute of Medical Research NIMR Nigerian Institute of Medical Research	@nimrnigeria	4,967

From the TAGS dataset we learnt two things. First, the dataset was not sufficient to answer the research question because of the query that was used and the limitation of the collection. It provided invaluable insight into setting up our main data collection and accounts to use, but many accounts did not include the #COVID19 hashtag in their tweets. Second, it allowed us to manually find active accounts across the world belonging to government administrations, public health agencies, and international non-governmental organizations. These accounts were searched on Twitter and ultimately included the main accounts of public health sectors, governments, and national leaders from 95 countries that are members of the International Association of National Public Health Institutes (IANPHI) (29). This list of accounts was used to extract data from the main study dataset.

#### GeoCov19 Dataset

The second dataset is a part of GeoCov19 collection by Qatar Computing Research Institute (QCRI). Messages were collected by using Twitter's Streaming API using hashtags, keywords, and geographical bounding boxes. The Artificial Intelligence for Disaster Response (AIDR) system was used for the data collection. Hundreds of different multilingual hashtags and keywords related to the COVID-19 pandemic were used (28). We used the list of accounts described in the previous section (TAGS dataset), and after removing inactive accounts, 133 were included, comprising 2,027,591 tweets authored by, replying to, or mentioning one of these accounts.

#### Combined Dataset

**Figure 1:**
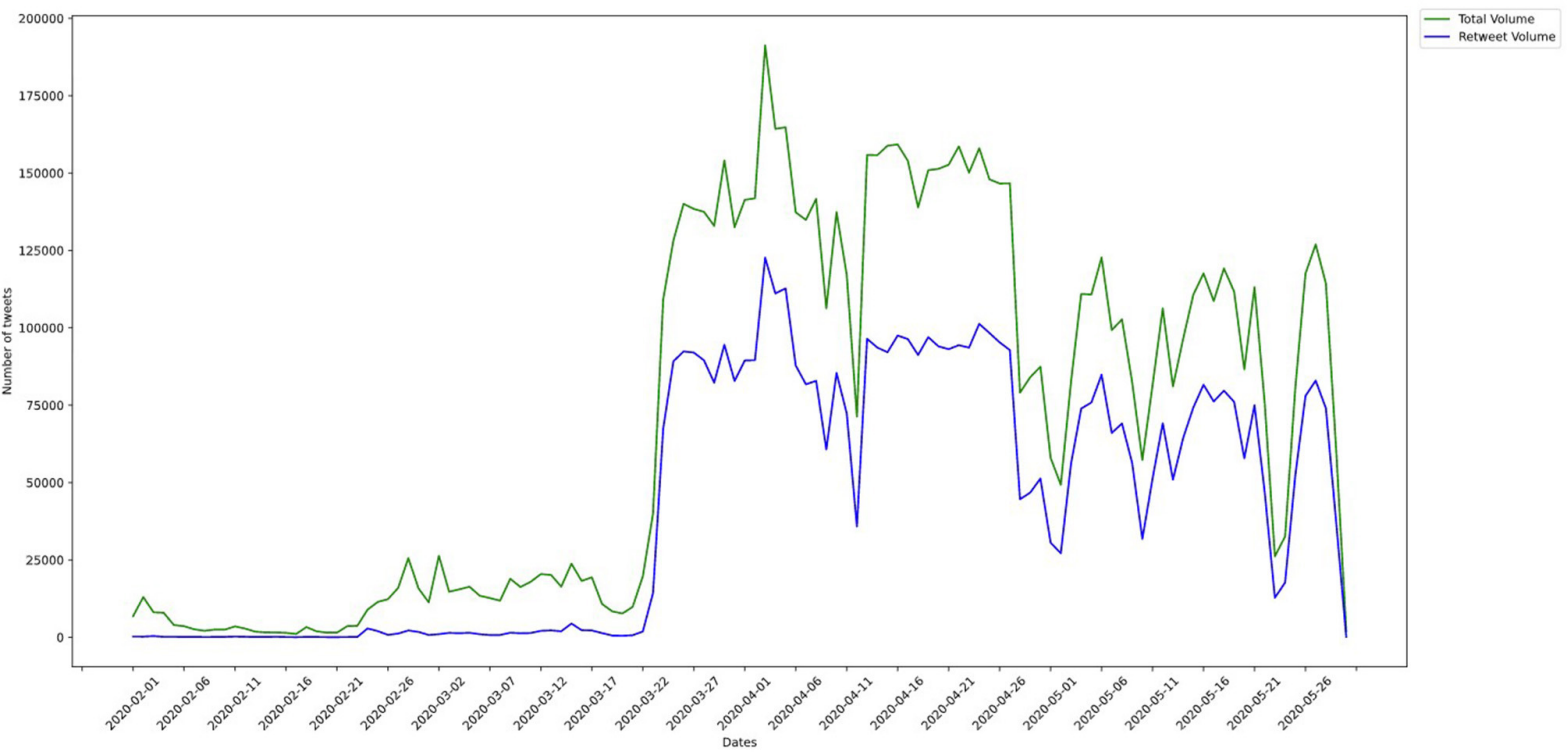
Time series of volume of tweets and retweets in combined dataset.

**Figure 2:**
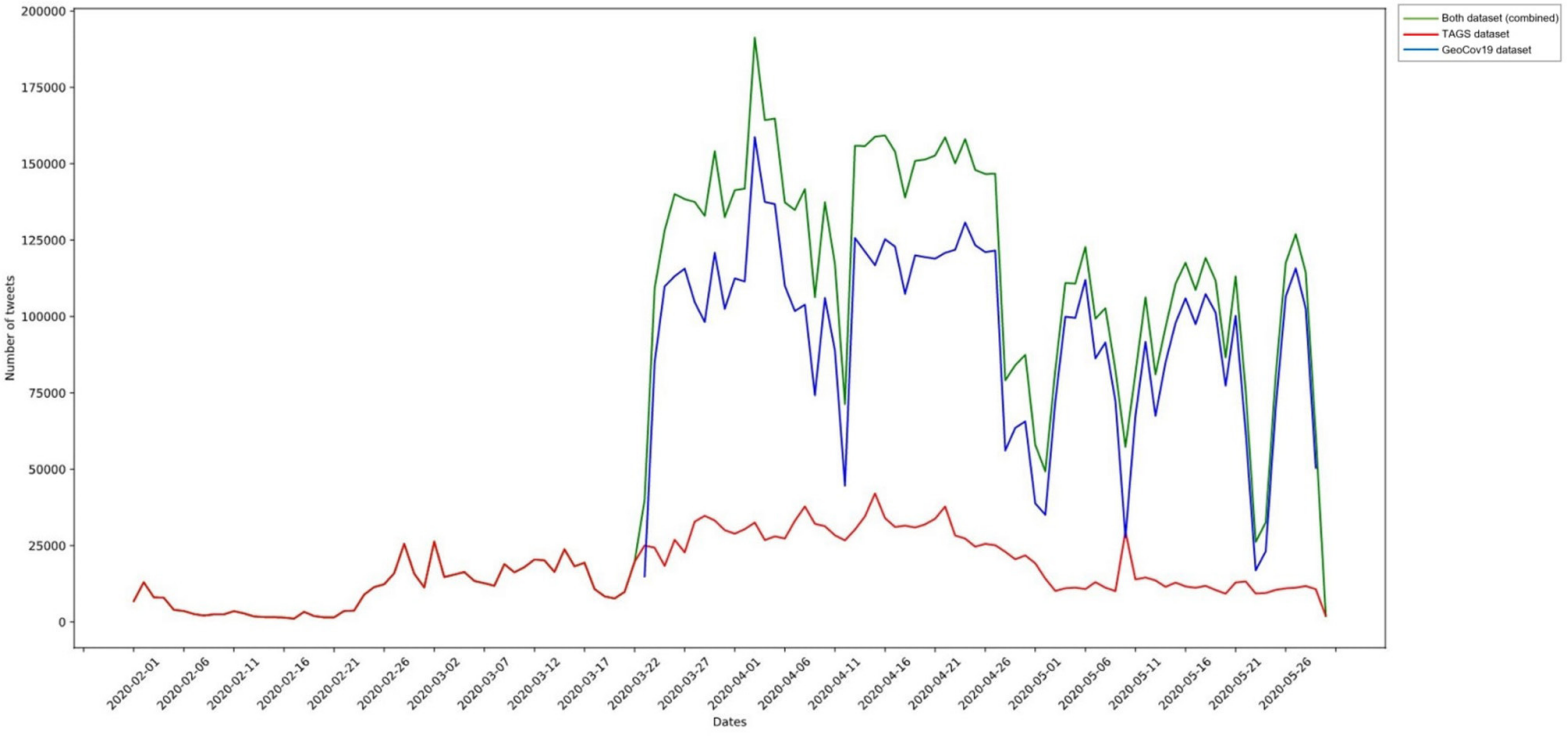
Time series of distribution of tweets in TAGS, GeoCov19, and combined datasets.

The final combined dataset (TAGS and GeoCov19) was used for carrying out the study. From the combined dataset, we selected the seven countries with the largest number of tweets for performing an in-depth analysis: Brazil, Canada, Spain, Mexico, Nigeria, UK and US. Figure 1 shows the overall volume of tweets and retweets separately. The other countries were excluded from the dataset due to the account availability (both accounts of the national government and public health agency in each country), geographic and language diversity and available tweets number of the account (minimum 1000 tweets). The overall distribution of analyzed tweets over time is shown in Figure 2, covering the period of 23 March 2020 to 29 May 2020.

The study investigated the UK in greater detail as the country's decentralized administration across four nations (England, Wales, Ireland, and Scotland) provided opportunities for dynamic risk communication challenges and varying local government messaging. In this further analysis, Wales was excluded based on the minimum number of tweets needed for analysis. A sufficient number of tweets to reliably perform automated clustering was collected from England, Scotland, and Northern Ireland. Table 1 shows the selected accounts and the total number of tweets for each.

### Data Analysis

First, we extracted the topics for each account to automatically cluster the data. Second, we extracted time series for comparing the activity of users and the words they used in different countries. Third, we made a qualitative analysis based on the collected materials. These steps were made because of the large volume of messages posted by users on Twitter, which made human annotation of each individual message impossible. This helped us to conduct the qualitative analysis.

#### Topic Modeling Through Automated Text Clustering

An unsupervised method was used to find topics by clustering messages based on their representation under the vector space model, using k-means as a clustering method (10).

Tweets were filtered by language. For each account analyzed, only tweets in the most popular language in that country were used in the dataset. Second, tweets were pre-processed by performing the same data cleaning procedure used in the word frequency analysis of previous section, and then features were extracted using TF-IDF vectorization (30).

**Figure 3:**
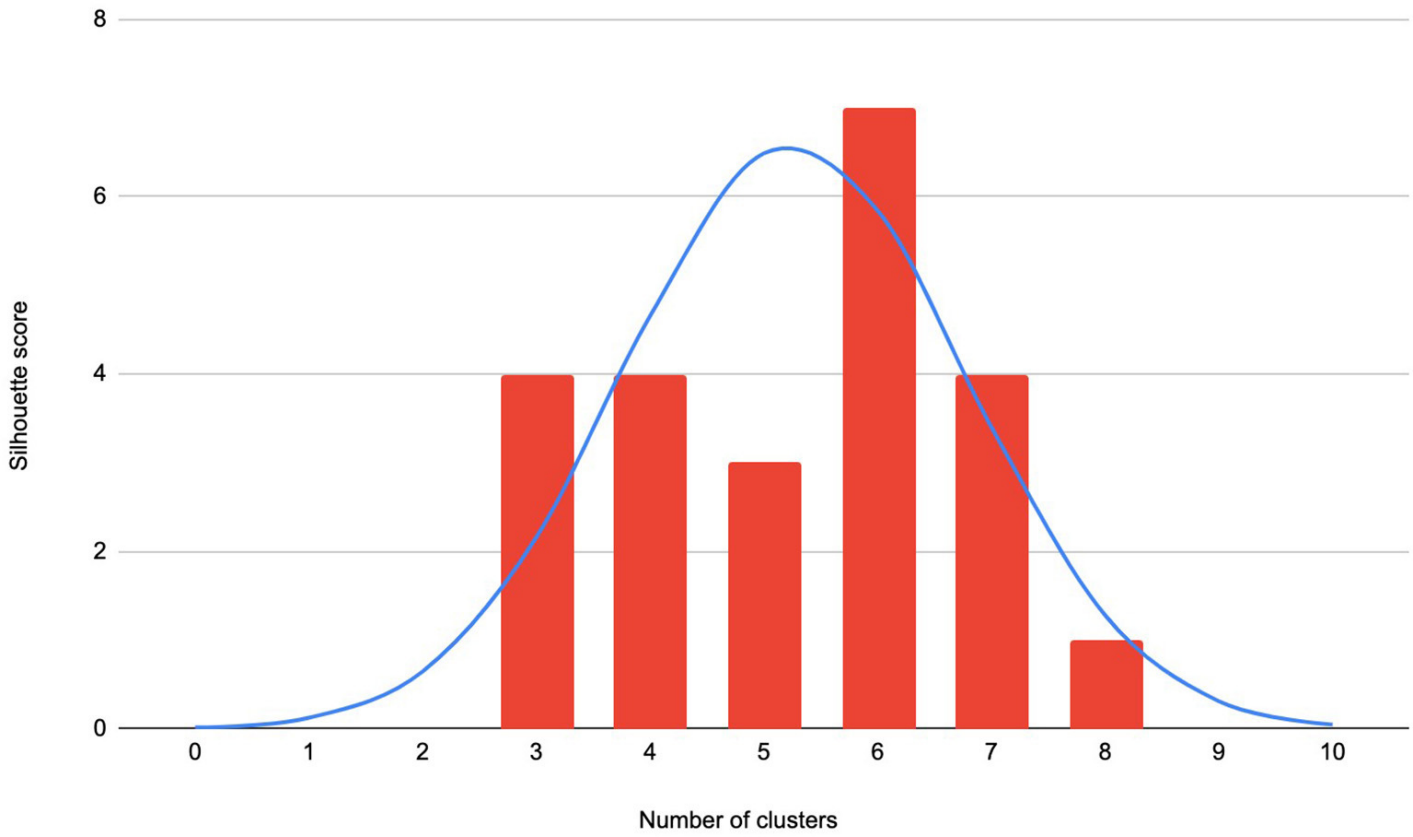
The distribution of the Silhouette scores of optimal k-values for analyzed accounts (A high Silhouette score for a number k in the x axis, means that the data can be coherently partitioned into k groups).

The k-means clustering algorithm was applied over these vectors using the Euclidean metric and 100 iterations. The number of clusters (k), were calculated using the Elbow and Silhouette methods. While the “elbow” method did not show clear results (it is a graphical method and plots generated on our data did not exhibit a clear “elbow”), the Silhouette method showed that the data can be clustered into 3–8 distinct groups. The distribution of Silhouette scores is shown in Figure 3. The blue line shows the distribution of Silhouette scores of different countries-related datasets.

Based on these results, messages were clustered into five topics for each selected account, resulting in 110 topics in total. For each of these topics, we computed the number of messages falling into that topic, its most frequent keywords, and a sample of messages that were frequently repeated (see Supplementary Material 1 for the full list).

#### Keywords and Time Series Analyses

Two simple characterizations of the data were performed to determine frequent keywords and frequency of postings over time. These characterizations could help public health experts to make qualitative analysis based on quantitative features of content. To determine frequent keywords, we removed sequences of digits, short words of less than three characters, common stop words (using the NLTK library) (31), and words that are simply COVID-19 topic markers such as “Coronavirus,”, “COVID19,” “COVID,” and “COVID-19.” We also removed hyperlinks, “@” signs from usernames, and non-Latin characters such as Arabic or Chinese characters, and implemented lower-casing, stemming, and tokenization (full code provided in Supplementary Material). The most frequent words were represented in a word cloud (see Supplementary Material 1). A time series analysis was performed to show the number of tweets in the dataset for each day within our observation window. Ultimately, both analyzes were carried out for each one of these accounts and the results are shown on figures in Supplementary Material 1.

#### Thematic Qualitative Analysis

**Figure 4:**
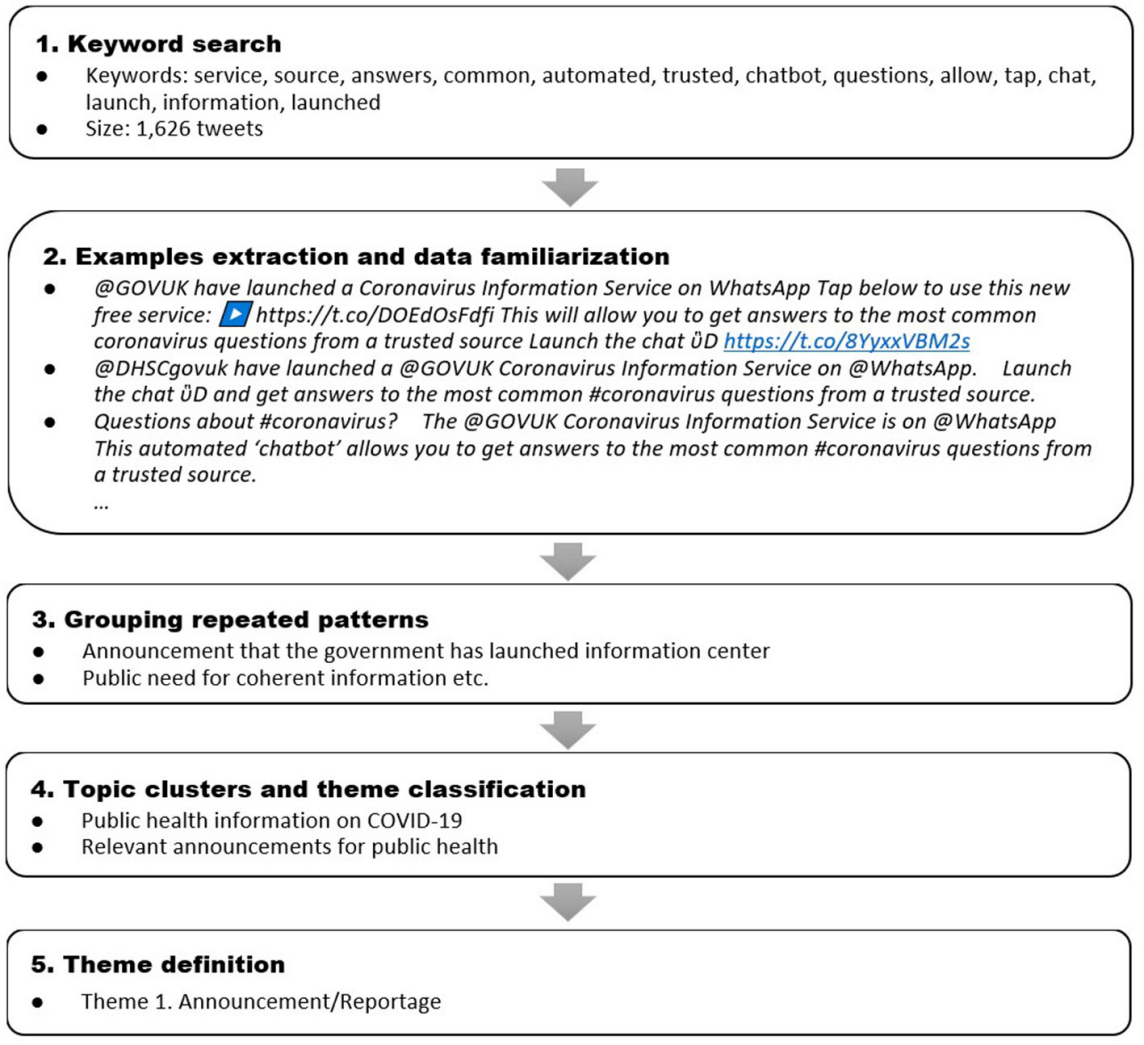
Flowchart displaying thematic classification process (Example from the @UK_gov account).

A six-steps approach for thematic analysis, developed by Braun and Clarke, was used to identify the main themes of the identified topics (32). Each of the 110 clusters created was closely examined by two senior public health experts (N.N., and C.C.) and one researcher (L.L.) for repeated topics and patterns of meaning (see Supplementary Material 2) and disagreements were resolved by consensus. Each topic was given a brief description, such as “Announcement that the government has launched an information centre,” which was possible in 103 of the clusters. Seven did not reflect any coherent topics that could be described and were filtered out. These descriptions were further reviewed to ensure they accurately represented the tweets in the cluster and were manually grouped in a bottom-up manner, into ten general themes (Table 2). The thematic analysis process is shown in the flowchart (Figure 4).

**Table 2:** Ten cluster themes and number of clusters and tweets in each theme.

**The final ten cluster themes identified (In no particular order)**	**No. of clusters**	**No. of tweets**
Announcement/Reportage: risk communication and dissemination of public health information	38	105,096
Combating misinformation and spread of fake news	5	6,353
Shortage of testing equipment, personal protective equipment (PPE)	6	58,340
Evidence-based leadership and policy for emergency response	19	622,462
Socio-economic consequences	10	346,344
Social measures (lockdowns, etc.)	8	79,818
Reproval on data equality: issues with data reporting and lack of inclusivity (gender, race, etc.)	5	8,479
Discrimination and vulnerable groups protection	5	4,582
Regional and global coordination or differences	4	1,238
Research-related topics (research gap, funding insufficiency)	3	7,814

Topic clusters were regrouped under their respective themes and the number of tweets in each theme for each country were counted. The top three most-discussed themes in each country were identified for making comparisons between different countries. In a similar manner as described above, the UK accounts were separated per nation (England, Scotland, and Northern Ireland, for which we had sufficient data) to produce a more detailed analysis including the differences between national and local governments.

## Results

In this section, we interpret the results of the thematic qualitative analysis and discuss the possible implications of the results for policy. We first describe the overall results based on the number of clusters and the number of tweets each contained and then compare the top themes among countries. Finally, the analysis of all included UK accounts has been analyzed as a case study to compare the differences between national and local policies.

### Overview

**Figure 5:**
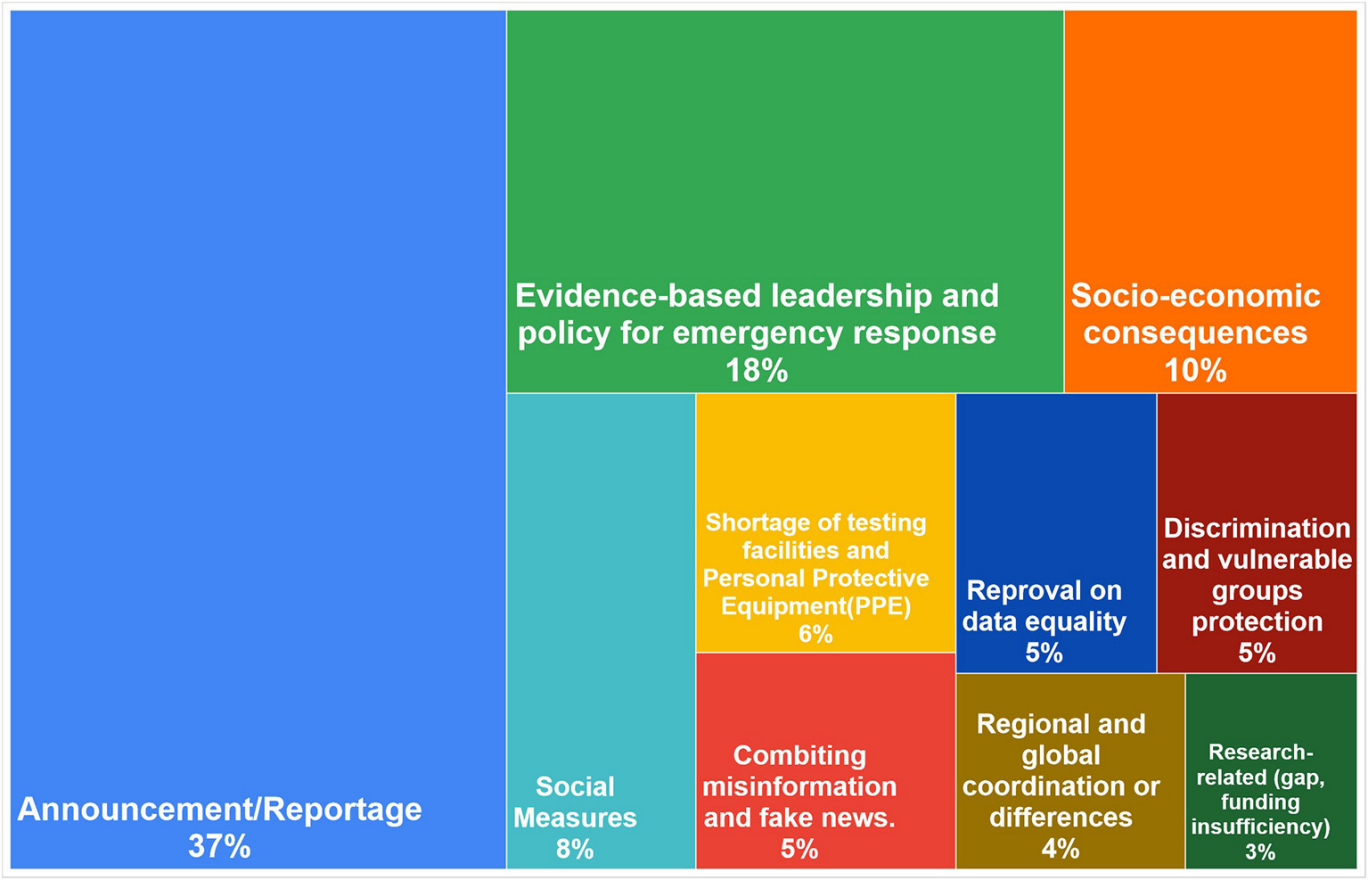
Distribution of the clusters in the 10 themes (the area represents the theme; the percentage represents the proportion of all clusters).

As shown in Figure 5, the most common theme (with the most topic clusters) was “announcement/reportage.” It accounted for 37% (38 clusters) of all topic clusters. This topic comprises announcements of public health information by the government or public health agencies. It also was the most-disseminated type of message by the analyzed accounts. This type of message included communication advising populations to follow government guidance or announcing new resources provided to support the public and ensure their safety, such as new information centres or testing facilities. Another common type of message within this theme is the reportage of the number of people being tested or found positive for COVID-19 and the daily mortality rate.

The second most frequent theme (19 clusters, 18%) was “evidence-based leadership and policy of emergency response.” This theme included broad criticism of governments, increasing public distrust of the governments' competence, and dissatisfaction with the way they handled the pandemic. It also included discontent and comments toward the policies and measures being implemented. The frequency of this theme reflects the discourse at the time regarding the need for strong, evidence-based leadership.

The third most frequent theme was “socio-economic consequences,” with 10 clusters (10%). The messages in this theme reflect the perception of the public that the economic policies implemented were not strong enough to meet the needs of the people, especially in the absence of financial protection.

Other themes were as follows in order of frequency: social measures (8%), shortage of testing facilities and personal protective equipment (PPE) (6%), combating misinformation and fake news (5%), reproval on data equality (5%), discrimination and vulnerable group protection (5%), coordination toward regional and global issues (4%), and research gaps and insufficient funding (3%).

### Comparison of Seven Countries

**Figure 6:**
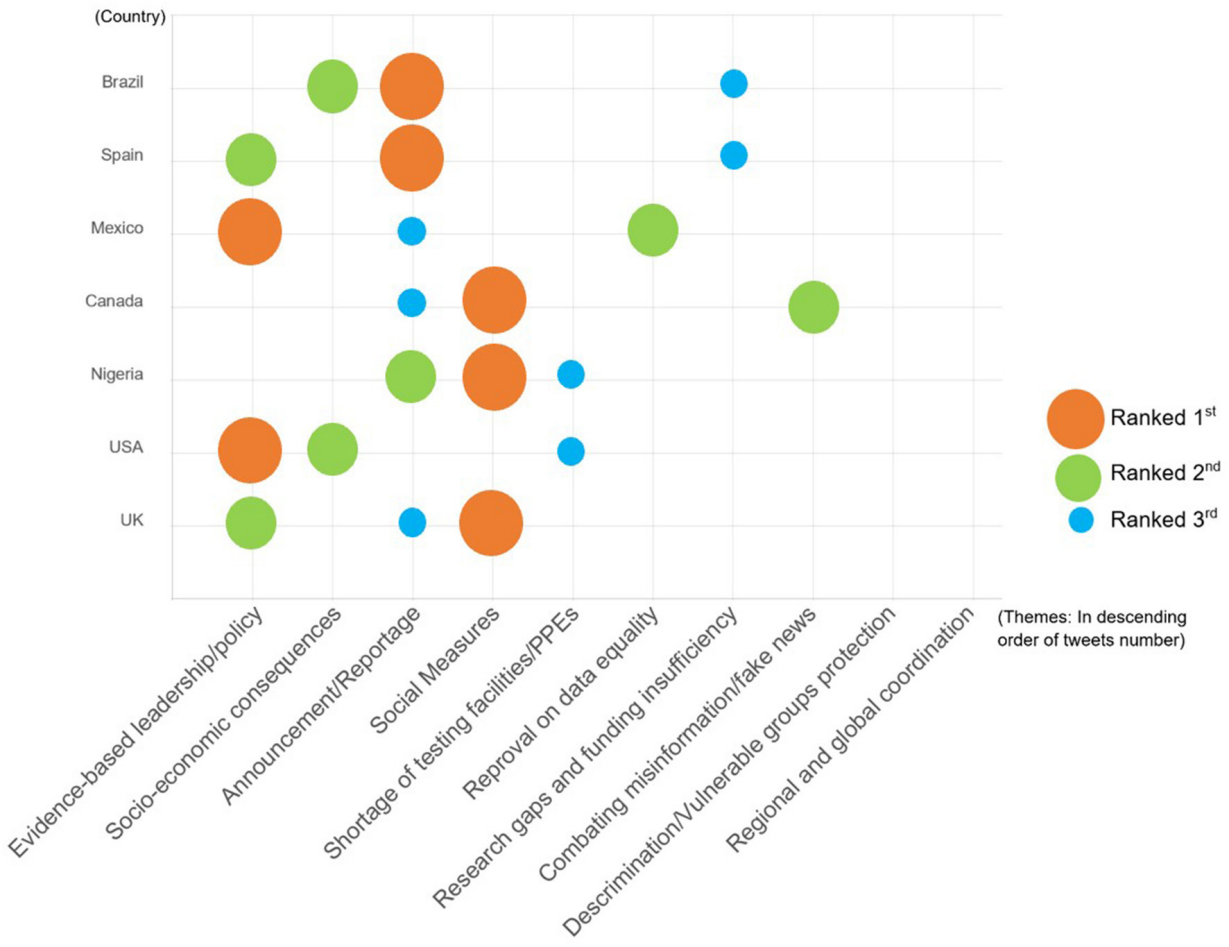
Top three themes per country.

The study compared all 10 themes in seven selected countries. From this comparison, we can see from Figure 6 that the rank of popularity across themes varied by country. In the US and Mexico, “evidence-based leadership/policy” ranked first while “social measures and lockdown” ranked higher in the UK, Nigeria, and Canada, and “announcement/reportage” in Spain and Brazil. However, even within the same topic, the focus of individual countries varied greatly due to different national conditions and policies.

#### Evidence-Based Leadership/Policy

The theme of evidence-based leadership and policy for emergency response ranked first in the US (56.7%) and Mexico (83.3%). Although the number of official accounts we selected varied from country to country, with the highest number being 8 in the UK, 3 in the US and Spain, and 2 in the rest, the US has had far more tweets than any other country, especially in this topic. A disproportionately high number of tweets were attributed to the former US President Donald Trump's Twitter account, most of which were politically related contents and discussions on evidences, as shown in the example tweets below. This finding is consistent with the previous studies (2, 33, 34).

^*^For the purpose of anonymity, all the tweet examples were paraphrased in this article.

“*US and South Korea had the first #COVID19 cases on the same day; within a week, South Korea approved a coronavirus test. The rollout of testing was a complete failure, allowing the virus to spread undetected.”*

“*As a doctor, @ ran vaccine programs to reduce the spread of the #coronavirus and to protect Americans from infection. The Democrats' attacks are baseless and just designed to stir up fear for political gain.”*

“*Who is this according to? The proof should be provided to us. Thousands of people have recovered from the #coronavirus with this treatment. So, your question was not a question at all, was it?”*

Similarly, in Mexico, one of the countries most affected by the COVID-19 pandemic, the most-discussed topic was also “evidence-based leadership” (83.3% of all Mexican tweets). We observed that most tweets in this topic were critical of the government response for various reasons, showing public discontent toward the government and leaders.

#### Social Measures

The second most discussed theme overall was that of “social measures.” The theme accounted for 85% of all tweets from the Canadian accounts in our sample. As of 17 March, 12 provinces and territories in Canada declared a state of emergency (35), and meanwhile, the Canadian government issued several social measures and gradually tightened them. The most prominent social measure in Canada was that of travel restrictions implemented since14 March 2020, which banned all unnecessary travel and only allowed Canadian citizens, permanent residents, and US citizens to come into the country (36, 37). However, 41% of COVID-19 cases reported were related to local transmission within Canada as of 21 March (38). According to the data collected, the restriction was met with both public scepticism and support online, shown in the sample tweet below.

“*Now is not the time to cross the border to visit a loved one in another country, especially if you are not a citizen or PR there. #Stayingathome and following government guidelines will help #flattenthecurve.”*

“*It's nonsense to lock down and ban the flights. Wake up, stand up for freedom! #stoplockdown”*

In the accounts from Nigeria, most of the social measures Tweets were related to the lockdown policy (63% of all Nigerian tweets). Following global practice, a nationwide lockdown was announced in Nigeria on 30 March 2020. However, the heavy economic costs of the lockdown in the country prompted the government to announce a phased and gradual easing of the lockdown, which resulted in a daily increase of case numbers in the de-escalation phase (39). The approach was also criticized for not following global practices and guidelines as stipulated by the WHO. During this period, the hashtag *#extendedlockdown* becoming a trending hashtag in Nigeria.

Similarly, in the UK the lockdown policies were also a target of public scrutiny on Twitter. However, as analyzed in this study, the policies adopted by the four UK nations differed, and most of the lockdown discussions were from the Scottish government, which got more positive comments compared with the other three nations.

#### Announcements and Reportage

The third most-discussed theme was the “announcement/reportage” category, which generally contained factual information, including the dissemination of public health information and general risk communication by government agencies. As shown in the Figure 6, this category ranked first in Brazil (66.3% of collected tweets) and Spain (60%), second in Nigeria (63%), and third in the UK (17.2%). Most of the content related to the multiple measures taken or announcements by each government to address the COVID-19 crisis and advocacy for the public to adhere to public health guidelines issued by the authorities, as shown in the example tweets below.

“*We have launched a Government Coronavirus Information Service on WhatsApp. Launch the chat and get answers to the most common #coronavirus questions from a trusted source.”*

“*We are out making sure our communities are kept safe this weekend. Follow the government guidelines: only leave home for shopping for food, household and medical supplies or travelling to & from work if you are a key worker”*

Our research data shows, in addition to the three most-discussed topics, the discussion on socio-economic consequences ranked the second in both the United States and Brazil, while the reproval of data equality and combating misinformation ranked the second in Mexico and Canada, respectively. Additionally, the shortage of protective equipment and testing facilities ranked third in both Nigeria and the United States. We also found that announcements and reports of research-related progress account for a large fraction of these messages in Spain and Mexico, including the study of vaccines and funding donated by other organizations.

### National Comparison: A Case Study of the UK

To explore the differences between policies set by national governments within one country, a case study of the UK was performed. A similar case study was explored in Brazil, known for its decentralized, state-run COVID response resulting from the federal government's handling of the pandemic; however, the volume of data was too limited and had too much noise to be analyzed adequately.

As mentioned, each of the included nations in the UK (England, Northern Ireland, Scotland) had autonomy for its own public health policy during the pandemic and independent Twitter accounts were available for data collection and analysis. We first examined the big picture and then analyzed the difference between each nation with evidence from policy.

**Figure 7:**
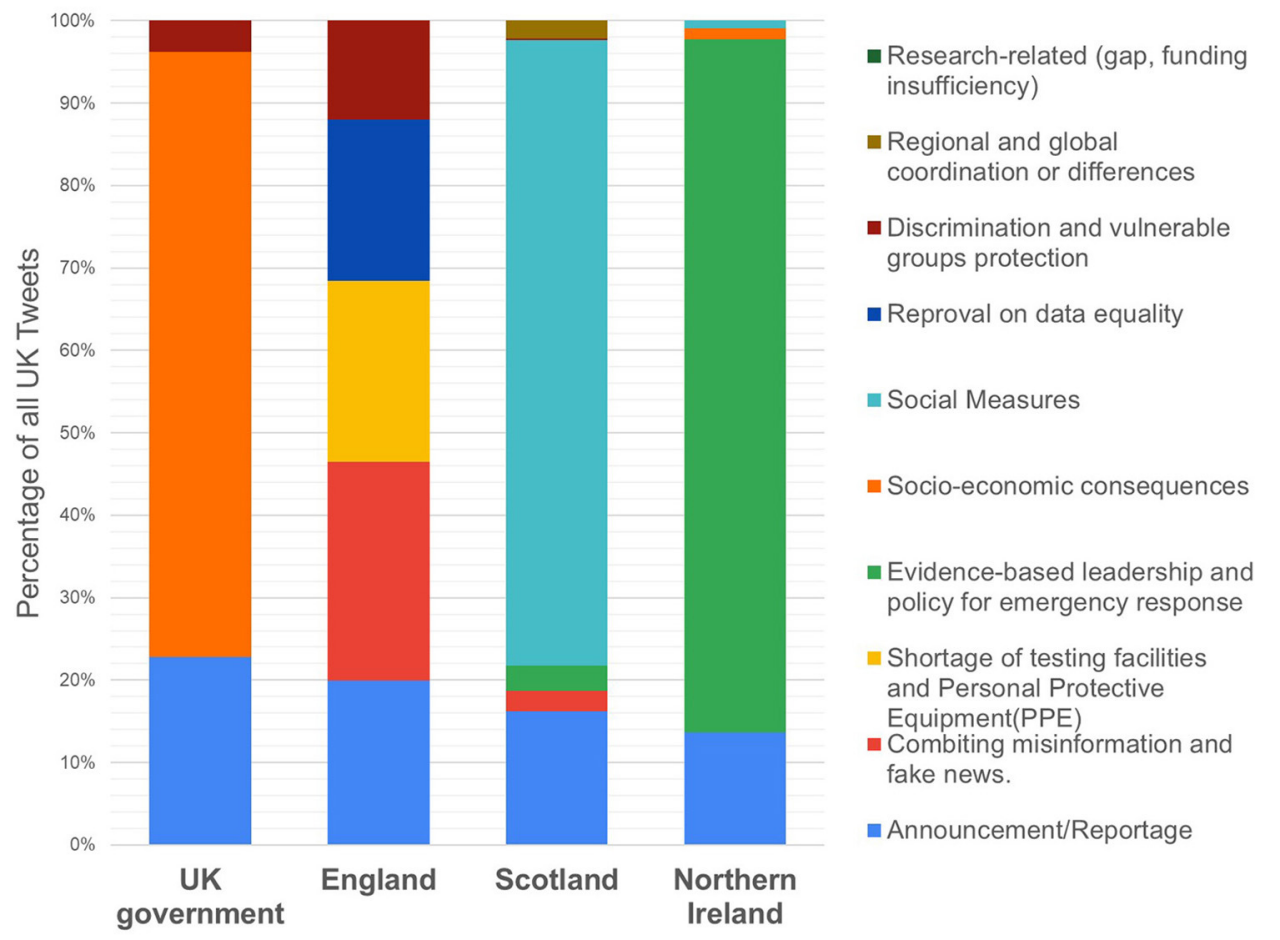
Themes discussed in each UK nation.

As shown in Figure 7, the content analysis revealed four key themes for the tweets including:

- Social measures/lockdown (30.22%): discussions on enforcement and adhering to public health and social measures and lockdowns.
- Evidence-based leadership/policy (27.97%): discontent and complaints toward leadership and policies.
- Announcement/reportage (17.08%): informational tweets disseminating public health information and announcements.
- Socio-economic consequences (16.09%): managing economic problems or discussions on the absence of financial protection.

The tweets included the UK government account (@govuk) were mostly about the “socio-economic consequences,” accounting for 73.3% of the tweets. This may be due to the announcement of an initial £330 billion package of emergency loan guarantees to help those in financial difficulty released on 17 March, followed by another round of fiscal support issued in an attempt to save UK businesses (40). However, comments frequently described the support package as “not well targeted” to save jobs in the most vulnerable industries such as in the retail, leisure, and hospitality sectors. According to the commentators, its practical effect was relatively limited and did not meet the specific needs of the country (41).

The case study revealed another major concern for those in the UK, that of the failure to gain “herd immunity.” UK government initially planned to tackle the pandemic by obtaining “herd immunity,” referring to “the reduction of infection or disease in the unimmunized segment as a result of immunizing a proportion of the population” (42, 43), This strategy spurred discussions on the policy direction, which included the debate on whether the UK should learn from Asian countries how to enforce social measures in the short term.

“*This so-called #HerdImmunity was completely a waste of time. We should have followed the approach of South Korea and continue to track and isolate every case when the #lockdown was imposed two weeks ago.”*

In addition to addressing the economic consequences, due to different policies and national conditions, each nation focused on specific concerns.

Social measures and lockdown (75% of all the Scottish tweets) were discussed frequently by people in Scotland, which is reflective of the policy in that nation during the time the data was collected. In mid-March, the UK nations diverged in their specific approaches toward lifting or reimposing restrictions based on epidemiological thresholds (44). In March, the Scottish government formed its own scientific advisory group to advise on the release of lockdown measures. Compared to other UK nations, the social restrictions in Scotland were more strict. This led to frequent tweets regarding easing lockdown toward the “New Normal” plans released by the government (45). However, social measures such as social distancing, self-isolation, and travel restrictions led to a reduced workforce across all economic sectors and caused job loss (46). Consequently, many people questioned the effects of the lockdown policy and its negative impact on the local economy. Fears of an impending economic crisis, such as a recession, were widely expressed on social media. Despite this, there was support for social restrictions to contain the outbreak, as shown in the example tweets below:

“*Coming out of lockdown is not the flick of a switch.” She told @ that while she wanted to end the lockdown as soon as possible, she also warned that it would become a ‘#NewNormal’ and those measures such as social distancing would remain.”*

“*We can repair the economy, but we can't bring back people who die.' She said she did not underestimate the economic damage of coronavirus, but that a premature end to the #lockdown could have a much greater impact on both lives and economies.”*

In Northern Ireland, the most frequently expressed sentiment was about the evidence-based leadership (83.6%), which related to political issues and the government's capacity and performance. As shown in the examples below, those criticisms were directed at UK ministers and politicians for not taking COVID-19 seriously enough in relation to existing evidence. Furthermore, we found that some political issues like party politics were also widely blamed for policy failures, as shown in the examples below:

“*Why don't we have politicians in Northern Ireland who can independently assess the knowledge, technology, engineering expertise and resources to fight the pandemic? We need to drop the slavish dogma, promote, and take benefit from our own expertise”*

“*The people who supported and voted for #BorisJohnson were disproportionately influenced by the coronavirus. It is not even callous self-interest for which he is notorious. This is insulting laziness and rudeness.”*

“*Unfortunately, @ @ @ made very regrettable comments tonight and @ should be working together rather than one party constantly undermining our confidence in our health system-we all deserve better”*

In addition, the most common criticism found in other studies (22, 23, 47), focussed on the slow and insufficient government response, requesting to learn from other successful countries such as China and South Korea, and blamed government departments for “buck-passing.”

Informative tweets also account for 14.3% of the total UK tweets that shared public health information or announcements. Most of them reported daily infection rates, announced new resources for supporting public health or advocated for adhering to government rules, and warned of penalties for violations.

## Discussion

In this section, we highlight the key findings, recommendations, and implications for practise and limitations of this work.

### Key Findings

Several findings emerged from this study. Firstly, social media has become a widely accepted channel for public health information and risk communication by government officials, public health agencies, and the general population. Secondly, in relation to our major study aim, this study found that the top three most-discussed topics during the COVID-19 pandemic were: (1) announcements of public health information, (2) requests for evidence-based leadership and policies, and (3) socio-economic consequences. Thirdly, the diverse political climate, national conditions, and cultural backgrounds contributed to the differences in popular content and themes across countries, which were mostly consistent with the local policy. This study also highlights the influence and function of key opinion leaders and political figures, which cannot be ignored given their frequent use of social media during outbreaks and other emergencies (48, 49).

### Implications for Policy and Practice

The widespread use of social media during the COVID-19 pandemic provides researchers an opportunity to understand communication themes from governmental and public health agencies, and the subsequent public discourse surrounding these communications.

Announcements and reportage were the most tweeted theme across countries. This is of note, as social media usage shifts away from solely recreational or promotional functions, the primacy of Twitter and other platforms as a risk communication and emergency announcement mechanism comes into focus.

It was made clear through this study that evidence-based policy and leadership in emergency response is frequently discussed, criticized, and questioned. This frequently discussed theme arises in tandem with the increase in public distrust of the governments' competence and dissatisfaction with their ways of handling the pandemic and measures implemented. Social media should be considered as an inevitable and major platform for governmental communications and subsequent feedback. Governmental and public health agencies are encouraged to maintain formal, evidence based, and consistent social media accounts, taking place of “town halls” from years past.

To improve this response in the digital era, the authorities should make full use of modern technology such as social media platforms to make the rationale for policy and decisions more visible, which shows transparency and could in turn increase trust by offering relevant and convincing health information to the public. Especially in a pandemic or in any large-scale emergency, it is vital for government and public health agencies' messaging to be consistent and aligned to avoid confusion and fear and to promote public trust and adherence to measures.

In addition, to better manage the negative socio-economic impacts of the pandemic, wide coverage and information equity are very important, especially for minority groups. Attention should be paid to the accessibility of social care and financial support to avoid a decline in credibility and public satisfaction caused by uneven distribution. Finally, social media can also be volatile: false information, sometimes maliciously created, spreads rapidly. Thus, measures to counter misinformation and disinformation on social media channels during an emergency are necessary. Given that government censorship can deeply aggravate already existing mistrust, measures other than content removal is needed, as the public shares and reacts positively to factual information, especially if posted by public health agencies.

### Limitations and Future Work

The strengths of this study lie in the multi-lingual and multi-country approach taken, as well as the use of a mixed approach. We collected data on the public's reactions to social media communications from government and public health agencies, which highlighted the importance of evidence-based policy, consistent information, and timely risk communication in handling a pandemic or other public health emergencies.

Several limitations in the review process and study design were identified. First, several layers of biases affect any information that can be gathered from social media, including sampling limitations in the public interfaces for data collection, demographic skews of social media populations, and the fact that we cannot identify the origin of each message, as people use automated accounts, disclose false locations in their profiles, and so on. These biases are something we tried to mitigate by comparing with government policies referred to in the social media messages.

Secondly, the number of messages originating from a country depends on several factors and does not necessarily reflect the public's interest on a topic. The consideration that Twitter users are younger, more technologically literate, and more likely to live in a city than general populations was also taken into account, among other demographic biases. Additionally, a message can become popular because it is posted by an account with many followers, not because it is intrinsically more relevant than any other message. However, in many cases, as the results section shows, the most popular topics often do reflect themes that capture the public's attention.

Thirdly, there were a few methodological challenges. This dataset collection did not naturally exhibit a clear number of clusters to be used when performing automated clustering, and while we did our best to examine this issue (Silhouette scores), we do not claim our choice of number of clusters is the only valid or useful one. The inevitable limitation is that since classification and merging of clusters is not completely automated, cognitive biases might affect the way in which we group categories of information although this task was undertaken by two senior experts in global and public health. It is unclear whether a fully automated method, such a machine learning classifier, would probably introduce different biases—but a participatory approach involving multiple perspectives might have produced a more robust grouping of information and better summaries.

## Conclusion

This study examines the roles of governments and public health agencies during the COVID-19 pandemic in seven countries and the public views on the policies implemented in each country by analyzing Twitter data through quantitative and qualitative methods. It is worth noting that this is a data-driven research supported by evidence instead of a policy evaluation or commentary between countries. We found that the main functions of these social media accounts during this phase of the pandemic were the expression of public opinions and the dissemination of health information. This content needs to be considered within specific national conditions and contexts, and there are differences that we found to be mostly consistent with local policies. Furthermore, evidence-based policy and leadership of emergency response is one of the most discussed topics across counties, which we found accompanied by an increase in public distrust of the government's competence and dissatisfaction with their way of handling the pandemic and measures implemented. Therefore, governments and public health departments need to enhance the transparency of policies and decisions to gain more public trust and to improve the public's support and compliance with public policies.

## Data Availability Statement

The datasets presented in this study can be found in online repository: https://github.com/vitiugin/covid_sm.

## Author Contributions

DN-O, CC, NN, and PK: conception or design of the work and final approval of the version to be published. AA and FV: data collection. LL, FV, AA, and NN: data analysis, interpretation, and drafting the article. NN, DN-O, CC, and PK: critical revision of the article. All authors contributed to the article and approved the submitted version.

## Funding

This research was partially supported by a research grant funded by the Belmont Foundation, UKRI (Reference number: NE/T013664/1). LL was partially supported by China Scholarship Council (File No. 202008060009). AA was funded by the Space and Aeronautics Research Institution, National Center for Satellite Technology, King Abdulaziz City for Science and Technology (KACST), Riyadh, Saudi Arabia. CC was partially supported by la Caixa Foundation (ID 100010434), under grant agreement LCF/PR/PR16/51110009. CC and FV were partially supported by project SoBigData++ funded by the EU under grant agreement 871042.

## Supplementary Material

The Supplementary Material for this article can be found online at: https://github.com/vitiugin/covid_sm

## Conflict of Interest

The authors declare that the research was conducted in the absence of any commercial or financial relationships that could be construed as a potential conflict of interest.

## Publisher's Note

All claims expressed in this article are solely those of the authors and do not necessarily represent those of their affiliated organizations, or those of the publisher, the editors and the reviewers. Any product that may be evaluated in this article, or claim that may be made by its manufacturer, is not guaranteed or endorsed by the publisher.
